# Exploratory mediation analysis of body roundness index in the nonlinear association between CHG index and metabolic dysfunction-associated steatotic liver disease among non-diabetic adults

**DOI:** 10.3389/fnut.2026.1868410

**Published:** 2026-07-23

**Authors:** Yanyan Xuan, Jingbo Yu, Qi Yao

**Affiliations:** 1 Department of Hospital Infection, The First Affiliated Hospital of Ningbo University, Ningbo, Zhejiang, China; 2 Department of Hepatology, The First Affiliated Hospital of Ningbo University, Ningbo, Zhejiang, China; 3 Department of Geriatrics Medicine, The First Affiliated Hospital of Ningbo University, Ningbo, Zhejiang, China

**Keywords:** Body roundness index, cholesterol, high-density lipoprotein, glucose, mediation analysis, metabolic dysfunction-associated steatotic liver disease, nonlinear relationship

## Abstract

**Background:**

Metabolic dysfunction-associated steatotic liver disease (MASLD) is the most prevalent chronic liver disease worldwide, highlighting an urgent need for non-invasive early biomarkers. The cholesterol, high-density lipoprotein, and glucose index (CHG) is a novel composite marker reflecting disturbances in glucose and lipid metabolism. However, the independent association between CHG and MASLD, and the potential statistical mediating effect of visceral adiposity, as represented by the body roundness index (BRI), remains unclear, especially in non-diabetic individuals.

**Methods:**

This cross-sectional study included 13,682 non-diabetic Japanese adults. Multivariable logistic regression, generalized additive models, and two-piecewise linear regression were used to explore the association, nonlinear relationship, and threshold effect between CHG and MASLD. Receiver operating characteristic (ROC) curve analysis was performed to evaluate CHG’s discriminatory performance. Exploratory mediation analysis was performed to statistically decompose the association between CHG and MASLD via BRI.

**Results:**

The prevalence of MASLD was 15.25%. After full adjustment, each 1-unit increase in CHG was independently associated with a higher risk of MASLD (OR = 4.46, 95% CI: 3.00–6.64). A significant dose–response trend was observed across CHG quartiles (Q4 vs. Q1: OR = 4.09, 95% CI: 2.83–5.91). A nonlinear threshold effect was identified at CHG = 5.52. CHG showed excellent discriminatory performance (AUC = 0.84), with an optimal cutoff at 5.23 (sensitivity = 0.81, specificity = 0.73), and retained robust discriminatory capacity in non-obese and young individuals. BRI showed a significant statistical mediating effect in the CHG–MASLD association, accounting for 46.15% of the total observed association. Subgroup mediation analyses revealed that this mediating effect was more pronounced in non-obese and normotriglyceridemic individuals.

**Conclusion:**

In non-diabetic Japanese adults, CHG is independently and nonlinearly associated with MASLD risk, with BRI as a potential statistical mediator. This mediating effect exhibits heterogeneity and is more pronounced in non-obese individuals. The causal direction of this pathway remains to be verified in prospective studies. CHG shows potential as a low-cost indicator for early MASLD risk stratification, especially in non-obese and younger populations who are often missed by conventional screening.

## 1 Introduction

Metabolic dysfunction-associated steatotic liver disease (MASLD) represents one of the most prevalent chronic liver diseases worldwide (1–3). The 2021 Global Burden of Disease study reported that MASLD affected 1.27 billion individuals, with an age-standardised prevalence of 15,018 per 100,000 population, and a particularly high disease burden in Asian populations (4–7). Based on the 2020 diagnostic framework and following the 2023 updated nomenclature consensus, MASLD is driven by systemic metabolic disorders and spans a spectrum from simple hepatic steatosis and metabolic dysfunction-associated steatohepatitis (MASH) to advanced liver fibrosis (8–10). As a leading cause of end-stage liver disease, MASLD is also closely linked to elevated risks of cardiovascular diseases, metabolic syndrome, and all-cause mortality (11–13). Due to its insidious onset and slow progression, early risk identification and targeted intervention are critical for slowing disease progression and improving prognosis.

Current strategies for MASLD screening and diagnosis remain substantially limited. Liver biopsy, the gold standard for pathology, is invasive and unsuitable for large-scale population screening (14, 15). Imaging methods such as ultrasound are limited by equipment and expertise, making them unsuitable for large-scale screening or long-term monitoring (16). Metabolic indices derived from routine blood biochemistry have become a research focus for early MASLD risk assessment due to their non-invasive, convenient, and scalable nature (17). The triglyceride-glucose index (TyG) is a commonly used marker of insulin resistance (IR) and has been independently associated with MASLD risk in multiple studies (18, 19). However, the TyG index captures only triglyceride and glucose metabolism, without accounting for the cholesterol homeostasis disorder that underlies MASLD pathogenesis. Although derived indices such as TyG-BMI have improved discriminatory performance to some degree, they perform poorly in non-obese individuals and tend to obscure the central features of metabolic dysfunction (20, 21).

The CHG (cholesterol, high-density lipoprotein, and glucose), first introduced by Mansoori et al. in 2025, integrates multidimensional metabolic signals from total cholesterol (TC), high-density lipoprotein cholesterol (HDL-C), and fasting blood glucose (FBG) (22). Compared with the TyG index, CHG not only reflects systemic IR but also captures impaired reverse cholesterol transport, a key pathogenic process (22–26). Hepatic cholesterol overload causes free cholesterol accumulation, inducing endoplasmic reticulum stress and mitochondrial dysfunction, and promoting hepatic steatosis (27, 28). Multiple prospective cohort studies have confirmed that CHG is strongly associated with cardiometabolic disease risk, with discriminatory performance similar to the TyG index but a distinct dose–response pattern, suggesting complementary clinical utility across different metabolic phenotypes (29, 30).

Despite these theoretical and practical strengths, the association between CHG and MASLD, as well as its underlying mechanisms, remains poorly understood. Most studies have not accounted for the confounding effect of diabetes, which, through associated metabolic disturbances, independently alters circulating metabolic markers and hepatic lipid deposition, thereby distorting the true relationship between CHG and MASLD (31, 32). The mediating role of visceral adiposity in the CHG-MASLD link remains unclear. The body roundness index (BRI), calculated from height and waist circumference (WC), more accurately reflects visceral fat distribution than body mass index (BMI) (33–36). While BRI has been linked to MASLD in a nonlinear manner, with stronger predictive power, no epidemiologic evidence supports the hypothesis that BRI mediates the pathway from CHG to MASLD (37, 38). Moreover, the value of CHG for risk stratification in non-obese and young individuals, often missed by conventional screening, remains unvalidated.

To fill these critical knowledge gaps, this study used the Japanese Non-Alcoholic Fatty Liver Disease (NAFLD) in the Gifu Area Longitudinal Analysis (NAGALA) cohort of non-diabetic adults to examine the independent association between CHG and MASLD, the mediating effect of BRI, and test the consistency of this association in non-obese and young subgroups. Our findings aim to provide new metabolic biomarkers and epidemiologic evidence for the non-invasive, early screening of MASLD.

## 2 Methods

### 2.1 Study design and population

We performed a secondary analysis using anonymised publicly available data from the NAGALA cohort. This population-based longitudinal health screening project was established in 1994 at Murakami Memorial Hospital in Gifu Prefecture, Japan. The NAGALA study was designed to investigate risk factors for metabolic disorders and chronic liver diseases, particularly type 2 diabetes mellitus (T2DM) and NAFLD, and to generate evidence for preventive public health strategies.

Since its establishment, participants have been enrolled in routine health examinations continuously. About 60% completed annual or biennial follow-up, yielding a high-quality longitudinal database. All data, including demographics, anthropometry, laboratory values, lifestyle factors, and medical histories, were collected by trained staff under standardised quality-controlled protocols to ensure data reliability. De-identified data were provided by Professor Takuro Okamura and deposited in the Dryad Digital Repository.^1^ The present study complied with the repository’s terms of service and data citation guidelines. The original NAGALA study was approved by the Ethics Committee of Murakami Memorial Hospital, and all participants provided written informed consent. Because we analysed only anonymised public data, no additional ethical approval was required under the Declaration of Helsinki. All procedures were performed in accordance with relevant ethical standards.

**Figure 1:**
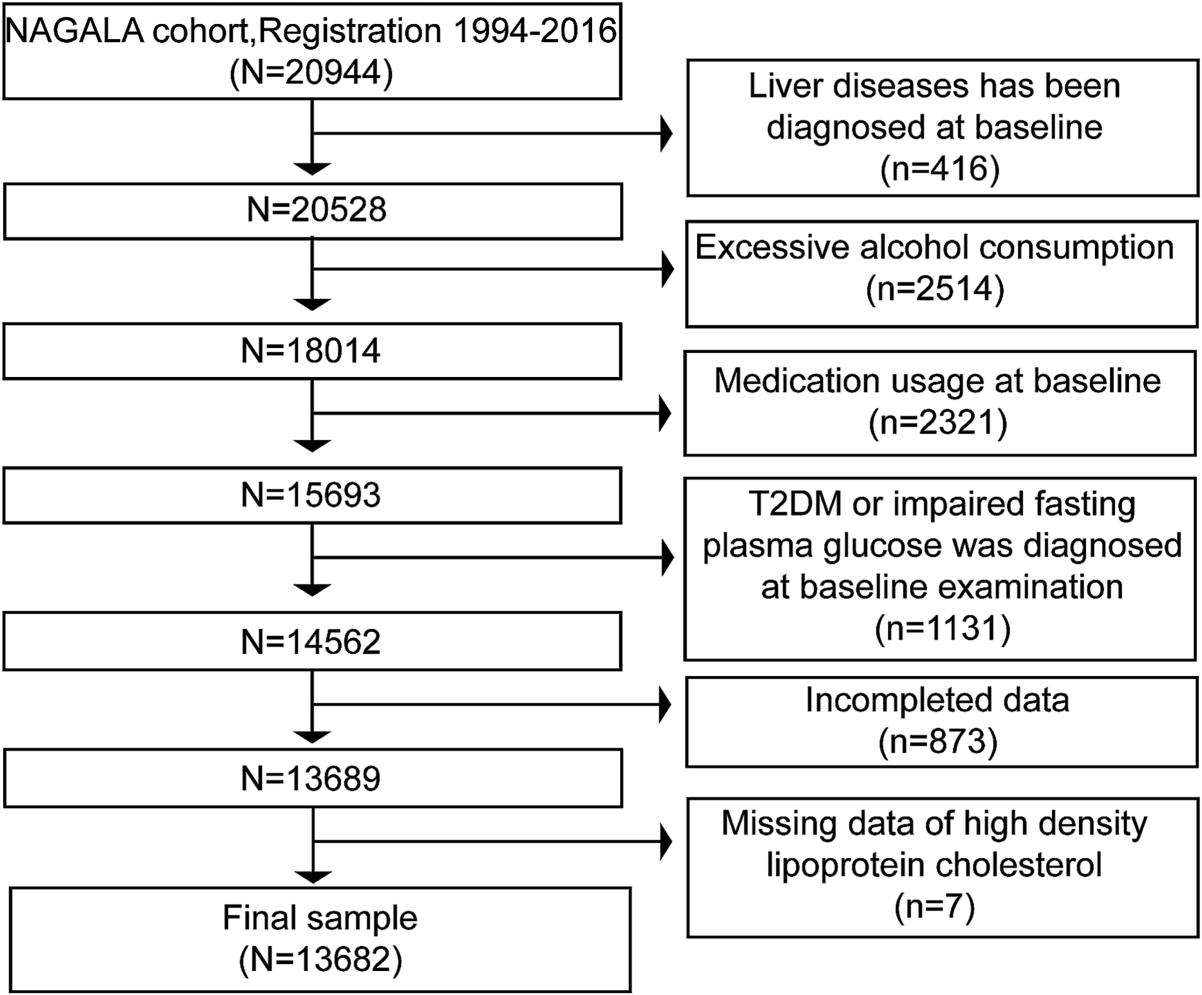
Flowchart of the study participants. NAGALA, Non-Alcoholic Fatty Liver Disease in the Gifu Area Longitudinal Analysis; T2DM, Type 2 diabetes.

A total of 20,944 adults who underwent health examinations between 1994 and 2016 were initially included. We applied a sequential exclusion process at baseline to reduce confounding: (1) chronic liver disease (*n* = 416); (2) excessive alcohol intake (>30 g/day for men, >20 g/day for women, *n* = 2,514); (3) regular long-term medication use (*n* = 2,321); (4) T2DM or impaired fasting glucose (*n* = 1,131); (5) missing key baseline data (*n* = 873); (6) missing HDL-C data (*n* = 7). After excluding 7,262 participants, 13,682 individuals remained in the final analysis. The participant flowchart is shown in Figure 1.

### 2.2 Definition of MASLD

Hepatic steatosis was diagnosed by abdominal ultrasonography performed by professionally trained technicians, and it is the essential pathological basis for the diagnosis of MASLD. The definitive diagnosis of hepatic steatosis was based on four internationally recognised ultrasonic features: increased hepatic parenchymal brightness, clear echogenic contrast between hepatic and renal cortices, blurring of intrahepatic vascular margins, and deep acoustic attenuation in hepatic parenchyma. All ultrasonic examinations were carried out in accordance with unified standardised operating protocols to ensure the consistency and reproducibility of diagnostic results across all participants.

MASLD was defined based on the core diagnostic framework from the 2020 International Expert Consensus Statement on metabolic dysfunction-associated fatty liver disease (originally termed MAFLD) (10). The updated nomenclature “metabolic dysfunction-associated steatotic liver disease (MASLD)” was adopted throughout the manuscript in accordance with the 2023 multisociety Delphi consensus statement on fatty liver disease renaming (8), which retained the full diagnostic criteria from the 2020 consensus with only terminology standardisation. The diagnosis of MASLD required the simultaneous satisfaction of two core conditions. First, hepatic steatosis was confirmed by histology, imaging, or validated blood-based biomarkers, with abdominal ultrasonography used for diagnosis in the present study. Second, participants had underlying metabolic abnormalities and met at least one of the following three criteria: (1) Overweight or obesity, defined by the Asian population-specific body mass index (BMI) cutoff of ≥23 kg/m^2^; (2) Confirmed diagnosis of T2DM in accordance with clinical diagnostic criteria; (3) Evidence of metabolic dysfunction, which was characterized by the presence of at least two abnormalities in the following cardiometabolic indicators: ① Abdominal obesity (Asian population-specific cutoffs: waist circumference ≥90 cm in men and ≥80 cm in women); ② Arterial hypertension (resting blood pressure ≥130/85 mmHg or current regular use of antihypertensive medications); ③ Hypertriglyceridemia-related dyslipidemia (serum triglyceride (TG) levels ≥1.7 mmol/L or current regular use of lipid-lowering agents); ④ Reduced serum HDL-C levels (HDL-C < 1.0 mmol/L in men and <1.3 mmol/L in women or current regular use of lipid-lowering agents); ⑤ Prediabetes FBG 5.6–6.9 mmol/L or glycated hemoglobin (HbA1c) 5.7–6.4%).

#### 2.2.1 Definition of CHG

CHG was determined using the following equation: ln[(TC × FBG)/(2 × HDL-C)], where TC represents total cholesterol (mg/dL), FBG is fasting blood glucose (mg/dL), and HDL-C is high-density lipoprotein cholesterol (mg/dL).

### 2.3 Definition of BRI

BRI was calculated as follows: BRI = 364.2–365.5 × (1 − [(WC/(2π))/(0.5 × height)]^2^)^0^·^5^ (33), where WC represents waist circumference (cm), and height denotes standing height (cm).

### 2.4 Variables

Study data were derived from the NAGALA database via standardised extraction procedures implemented to maintain the uniformity and reliability of all assessed indicators. Trained investigators delivered a validated structured questionnaire to collect participants’ basic demographic information (age, sex), anthropometric measures (height, weight, WC), and lifestyle behaviours (smoking habit, alcohol intake, exercise status). All anthropometric tests were performed by qualified technicians using calibrated standard equipment, in accordance with the operational protocols of the original NAGALA cohort. Lifestyle information was acquired through standardised face-to-face interviews, in accordance with the cohort’s established methodological criteria.

Venous blood specimens were collected from all participants after a minimum 8-h overnight fast. Biochemical tests were analysed using an automated clinical biochemistry analyser with rigorous quality assurance protocols. The measured biochemical indicators included serum lipid profiles (TC, HDL-C, low-density lipoprotein cholesterol [LDL-C], TG), glycemic control markers (FBG, HbA1c), and hepatic function enzymes (aspartate aminotransferase[AST], alanine aminotransferase [ALT], Gamma-glutamyl transpeptidase [GGT]).

According to self-reported smoking history, participants were divided into three distinct groups: never smokers, ex-smokers, and current smokers. Exercise status was stratified into two categories: regular exercisers (those with at least 1 session of moderate-to-vigorous exercise per week) and inactive individuals. Hypertension was defined according to the 2019 Guidelines of the Japanese Society of Hypertension (JSH), as office systolic blood pressure (SBP) ≥140 mmHg and/or diastolic blood pressure (DBP) ≥90 mmHg, or regular antihypertensive drug use at baseline (39). BMI was calculated as weight (kg) divided by the square of height (m^2^).

The primary endpoint of this study was MASLD. The CHG index served as the main independent variable, and BRI was the mediating variable. Covariates were selected based on prior clinical and epidemiological research on MASLD. Continuous covariates included age, BMI, LDL-C, TG, AST, ALT, GGT, and HbA1c, and categorical covariates included sex, smoking status, alcohol consumption, exercise status, and hypertension status.

### 2.5 Statistical analysis

All data processing and statistical analyses were performed using R software (version 4.1.0, https://www.R-project.org) and EmpowerStats (version 4.0, https://www.empowerstats.com). Baseline characteristics of participants were described according to quartiles of the CHG index. Continuous variables were presented as mean ± standard deviation for normally distributed data or median (interquartile range) for skewed data; categorical variables were expressed as percentages. Univariate and multivariate logistic regression models were used to examine the association between CHG and MASLD, with three models constructed: an unadjusted model without covariate adjustment, a minimally adjusted model adjusted only for age and sex, and a fully adjusted model incorporating age, sex, hypertension, smoking status, drinking status, exercise status, HbA1c, BMI, ALT, AST, GGT, TG and LDL-C.

Covariates were selected *a priori* based on established clinical and epidemiological evidence of well-recognized confounders for MASLD, including demographic characteristics, lifestyle factors, metabolic biomarkers, and liver enzymes. Missing data were handled using complete case analysis. Participants with missing data on key variables (CHG, MASLD status, or core covariates) were excluded from the final analysis. The overall missing rate was low, and missing data were considered to occur randomly; thus, complete case analysis would not introduce substantial bias.

To verify the robustness of the primary findings across different populations, sensitivity analyses were conducted. Stratified analyses were performed by sex, age group, BMI, WC, TG categories, and hypertension status using multivariate logistic regression, and the consistency of statistical results across these subgroups was compared. In addition, generalised additive models (GAMs) were applied to explore potential nonlinear relationships between CHG and MASLD risk. GAM enables flexible modelling of complex relationships without prespecifying functional forms, accommodating different data types and interactions while reducing the risk of overfitting. Based on the GAM results, smoothing curves for the nonlinear relationship were plotted, and a two-piece linear regression model derived from these curves was used to estimate the effect of the CHG threshold on MASLD risk.

Receiver operating characteristic (ROC) curve analysis was performed to evaluate the discriminatory performance of CHG and other biochemical parameters for MASLD. DeLong’s test was used to compare differences in the area under the ROC curve (AUC) between CHG and other indicators. The optimal cutoff value of CHG was determined by maximizing Youden’s index (sensitivity + specificity − 1), which balances diagnostic sensitivity and specificity. Internal validation of the AUC and cutoff estimates was performed via 1,000 bootstrap resampling replicates to derive empirical 95% confidence intervals and assess result stability. Statistical significance was defined as a two-sided *p*-value < 0.05.

Given the cross-sectional study design, we performed exploratory mediation analysis to statistically decompose the observed association between CHG and MASLD, with BRI as the potential mediator. This analysis is intended for descriptive statistical inference only and does not imply a causal pathway. To explore the statistical mediating effect of BRI in the association between CHG and MASLD risk, we first used a multiple linear regression model to analyse the relationship between CHG and BRI. Subsequently, multiple logistic regression was adopted to examine the association between BRI and MASLD risk. Finally, the “mediation” package in R was used to perform mediation-effect analysis to evaluate the direct and indirect components of the observed association between CHG and MASLD via BRI. Specifically, we followed the standard method of the “mediation” package, which estimates mediation effects through a two-step procedure: the first step was to construct a mediation model with BRI as the mediator, and predict the changes of BRI under the influence of CHG and other covariates through regression analysis; the second step was to establish an outcome model to assess the risk of MASLD based on CHG and the mediating variable BRI, with potential confounding factors controlled for.

For the core mediation model, covariates included age, sex, hypertension, smoking status, alcohol consumption, regular exercise, HbA1c, ALT, AST, GGT, TG, and LDL-C. BMI was not adjusted for in the primary analysis, given the high correlation between BMI and BRI (the mediator). This approach was adopted to avoid collinearity-induced over-adjustment and potential underestimation of the indirect effect, as BRI already sufficiently captures body fat distribution and visceral adiposity, which is the core pathophysiological pathway of interest in this study.

To evaluate the potential impact of incorporation bias due to overlapping metabolic components between the exposure/mediator and MASLD diagnostic criteria, we performed a sensitivity analysis using ultrasound-diagnosed isolated hepatic steatosis (without metabolic dysfunction criteria) as the outcome. The core association and mediation analyses were repeated with the same covariate sets as the primary analyses to verify the robustness of the main findings.

To quantify the mediating effect, the direct effect of CHG on MASLD risk (i.e., the effect independent of BRI) and the indirect effect (i.e., the effect mediated by BRI) were separately calculated. The “mediation” package provides the average causal mediation effect, which reflects the average indirect effect of CHG on MASLD risk through BRI, and the average direct effect, which represents the portion of CHG’s direct effect on MASLD risk that BRI does not modulate. The ratio of the indirect effect to the total effect quantified the mediation effect’s magnitude. When the total, direct, and indirect effects were in the same direction, the mediation proportion was calculated to quantify the fraction of the total effect attributable to the mediating variable BRI.

To ensure the accuracy of the analysis results, 1,000 Bootstrap resampling tests were conducted, and the significance of the mediation effect was determined by a 95% confidence interval (CI). Covariates were adjusted in all calculations to minimise the impact of confounding factors. A mediation effect was considered statistically significant if the 95% CI did not include zero. Therefore, a significant mediation effect was confirmed only when three conditions were met simultaneously: a significant indirect effect, a significant total effect, and a positive mediation proportion. A two-tailed *p*-value < 0.05 was considered statistically significant for all analyses.

## 3 Results

### 3.1 Study population demographic and features

A total of 13,682 participants were included in the present study, of whom 2,087 were diagnosed with MASLD—corresponding to a population-level prevalence of 15.25%. The study population had a mean age of 43.38 ± 8.84 years, and the sex distribution was 50.91% male and 49.09% female. CHG levels were normally distributed, ranging from 4.03 to 6.40, with a mean (standard deviation) of 5.10 (0.34). Anthropometric and biochemical characteristics of participants, stratified by CHG quartiles, are summarised in Table 1. We observed that multiple clinical and laboratory parameters—age, BMI, body weight, height, WC, SBP, DBP, FBG, HbA1c, TC, LDL-C, BRI, ALT, AST, GGT, and TG were significantly elevated with increasing CHG concentrations (all *p* < 0.001). Conversely, HDL-C showed an inverse trend. As CHG quartiles increased, the proportions of female participants, subjects aged ≥40 years, individuals with BMI ≥ 25 kg/m^2^, ever or current smokers, and those with MASLD or hypertension rose progressively. In contrast, the percentages of male participants, subjects aged <40 years, individuals with BMI < 25 kg/m^2^, never smokers, and those without hypertension or MASLD declined gradually with ascending CHG levels (all *p* < 0.001).

**Table 1:** Baseline characteristics of the study population according to the CHG quartile group.

Characteristic	CHG quartile				*p*-value
	Q1 (4.03–4.85)	Q2 (4.85–5.08)	Q3 (5.08–5.34)	Q4 (5.34–6.40)	
No. of participants	3,421	3,420	3,420	3,421	
Age (years, mean ± SD)	40.46 ± 8.08	42.70 ± 8.54	44.73 ± 9.08	45.62 ± 8.74	<0.001
Age (years, N, %)					<0.001
<40	1708 (49.93%)	1,355 (39.62%)	1,120 (32.75%)	974 (28.47%)	
≥40	1713 (50.07%)	2065 (60.38%)	2,300 (67.25%)	2,447(71.53%)	
Sex (*N*, %)					<0.001
Male	2,828 (82.67%)	2,166 (63.33%)	1,347 (39.39%)	625 (18.27%)	
Female	593 (17.33%)	1,254 (36.67%)	2073 (60.61%)	2,796 (81.73%)	
Smoking behaviour (*N*, %)					<0.001
Never smoke	2,750 (80.39%)	2,461 (71.96%)	1951 (57.05%)	1,488 (43.50%)	
Ever smoke	385 (11.25%)	476 (13.92%)	690 (20.18%)	796 (23.27%)	
Current smoke	286 (8.36%)	483 (14.12%)	779 (22.78%)	1,137 (33.24%)	
Drinking behaviour (*N*, %)					<0.001
Never/light drink	2,997 (87.61%)	2,982 (87.19%)	2,885 (84.36%)	2,938 (85.88%)	
Moderate drink	351 (10.26%)	405 (11.84%)	519 (15.18%)	479 (14.00%)	
Heavy drink	73 (2.13%)	33 (0.96%)	16 (0.47%)	4 (0.12%)	
Hypertension (*N*, %)	58 (1.70%)	107 (3.13%)	208 (6.08%)	363 (10.61%)	<0.001
Habit of exercise (*N*, %)	600 (17.54%)	630 (18.42%)	571 (16.70%)	545 (15.93%)	0.040
BMI (kg/m^2^, mean ± SD)	19.98 ± 2.21	21.17 ± 2.53	22.61 ± 2.92	24.30 ± 3.10	<0.001
BMI (kg/m^2^, *N*, %)					<0.001
<25	3,340 (97.63%)	3,189 (93.25%)	2,832 (82.81%)	2,172 (63.49%)	
≥25	81 (2.37%)	231 (6.75%)	588 (17.19%)	1,249 (36.51%)	
Weight (kg, mean ± SD)	52.05 ± 7.85	56.49 ± 9.29	62.23 ± 10.54	68.72 ± 11.19	<0.001
Height (cm, mean ± SD)	161.19 ± 7.36	163.09 ± 8.33	165.61 ± 8.68	167.92 ± 7.93	<0.001
WC (cm, mean ± SD)	69.48 ± 6.62	73.14 ± 7.49	77.93 ± 8.03	83.10 ± 8.00	<0.001
SBP (mmHg, mean ± SD)	107.09 ± 13.02	110.82 ± 13.47	115.93 ± 14.15	120.54 ± 14.80	<0.001
DBP (mmHg, mean ± SD)	66.10 ± 9.18	68.83 ± 9.38	72.44 ± 9.81	76.03 ± 10.09	<0.001
ALT (IU/L, median, quartile)	14.00 (11.00–17.00)	15.00 (11.00–19.00)	17.00 (13.00–23.00)	23.00 (16.00–32.00)	<0.001
GGT (IU/L,median, quartile)	12.00 (10.00–15.00)	13.00 (10.00–17.00)	15.00 (12.00–21.00)	20.00 (15.00–29.00)	<0.001
AST (IU/L, median, quartile)	16.00 (13.00–19.00)	16.00 (13.00–20.00)	17.00 (14.00–21.00)	19.00 (15.00–23.00)	<0.001
TG (mmol/L, median, quartile)	0.45 (0.34–0.60)	0.60 (0.44–0.79)	0.80 (0.61–1.07)	1.30 (0.95–1.81)	<0.001
TC (mmol/L, mean ± SD)	4.69 ± 0.75	4.94 ± 0.77	5.20 ± 0.79	5.65 ± 0.86	<0.001
HDL-C (mmol/L, mean ± SD)	1.89 ± 0.36	1.57 ± 0.27	1.34 ± 0.23	1.07 ± 0.19	<0.001
LDL-C (mmol/L, mean ± SD)	2.58 ± 0.53	3.08 ± 0.55	3.46 ± 0.61	3.90 ± 0.79	<0.001
FBG (mmol/L, mean ± SD)	4.86 ± 0.36	5.05 ± 0.36	5.22 ± 0.36	5.41 ± 0.35	<0.001
HbA1c (%, mean ± SD)	5.11 ± 0.29	5.15 ± 0.31	5.20 ± 0.32	5.27 ± 0.34	<0.001
BRI (mean ± SD)	2.19 ± 0.66	2.49 ± 0.76	2.88 ± 0.87	3.31 ± 0.89	<0.001
MASLD (*N*, %)	38 (1.11%)	145 (4.24%)	499 (14.59%)	1,405 (41.07%)	<0.001

Mean ± SD or medians (quartiles) were for continuous variables. % was for categorical variables.

MASLD, Metabolic dysfunction associated steatotic liver disease; CHG, Cholesterol, high-density lipoprotein, and glucose; BMI, Body mass index; WC, Waist circumference; SBP, Systolic blood pressure; DBP, Diastolic blood pressure; ALT, Alanine aminotransferase; AST, Aspartate aminotransferase; GGT, Gamma-glutamyl transpeptidase; HbA1c, Glycosylated hemoglobin; FBG, Fasting blood glucose; TG, Triglycerides; TC, Total cholesterol; HDL-C, High-density lipoprotein cholesterol; LDL-C, Low-density lipoprotein cholesterol; BRI, Body roundness index.

### 3.2 Association between CHG and MASLD

To investigate the association between CHG and MASLD risk, multivariate logistic regression analyses were performed; the results are presented in Table 2. CHG was positively associated with MASLD risk across all three regression models (all *p* < 0.001). In the unadjusted Model 1, each unit increase in CHG was associated with an odds ratio (OR) of 74.71 (95% confidence interval [CI]: 61.61–90.59) for MASLD. After adjusting for age and sex (Model 2), this association remained significant, with an OR of 52.66 (95% CI: 42.71–64.93). In the fully adjusted Model 3 (accounting for potential confounders), the OR was 4.46 (95% CI: 3.00–6.64), confirming the independent positive association between CHG and MASLD risk. When CHG was categorised into quartiles (Q1: 4.03–4.85, Q2: 4.85–5.08, Q3: 5.08–5.34, Q4: 5.34–6.40), with Q1 as the reference group, a significant dose–response relationship was observed across all models. Specifically, the ORs for Q2, Q3, and Q4 were 3.94, 15.21, and 62.04 in Model 1; 3.40, 11.28, and 41.05 in Model 2; and 1.74, 2.64, and 4.09 in Model 3, respectively, indicating that higher CHG quartiles were progressively associated with increased MASLD risk.

**Table 2:** Associations between CHG and the MASLD risk.

Variable	Model 1 OR (95% CI), *p*-value	Model 2 OR (95% CI), *p*-value	Model 3 OR (95% CI), *p*-value
CHG	74.71 (61.61, 90.59) < 0.001	52.66 (42.71, 64.93) < 0.001	4.46 (3.00, 6.64) < 0.001
Q1 (4.03–4.85)	Reference	Reference	Reference
Q2 (4.85–5.08)	3.94 (2.75, 5.65) < 0.001	3.40 (2.37, 4.88) < 0.001	1.74 (1.17, 2.61) 0.007
Q3 (5.08–5.34)	15.21 (10.90, 21.23) < 0.001	11.28 (8.03, 15.85) < 0.001	2.64 (1.79, 3.91) < 0.001
Q4 (5.34–6.40)	62.04 (44.74, 86.04) < 0.001	41.05 (29.30, 57.53) < 0.001	4.09 (2.69, 6.23) < 0.001
*p* for trend	<0.001	<0.001	<0.001
Subgroup analysis			
Sex			
Men	91.27 (61.74, 134.91) <0.001	87.27 (57.85, 131.65) <0.001	3.37 (1.51, 7.51) 0.029
Women	39.31 (30.96, 49.90) <0.001	41.79 (32.80, 53.26) <0.001	4.59 (2.89, 7.28) <0.001
Age (years)			
<40	164.35 (113.55, 237.86) <0.001	109.55 (73.67, 162.90) <0.001	3.95 (1.80, 8.64) <0.001
≥40	53.61 (42.63, 67.42) <0.001	35.27 (27.63, 45.02) <0.001	4.82 (3.02, 7.69) <0.001
BMI (kg/m^2^)			
<25	69.85 (53.65, 90.94) <0.001	39.69 (29.72, 52.99) <0.001	5.83 (3.52, 9.65) <0.001
≥25	11.21 (7.95, 15.80) <0.001	8.45 (5.87, 12.16) <0.001	2.49 (1.31, 4.71) 0.005
TG (mmol/L)			
<1.7	63.33 (50.52, 79.40) <0.001	41.48 (32.45, 53.03) <0.001	8.19 (5.48, 12.25) <0.001
≥1.7	9.98 (5.82, 17.10) <0.001	10.81 (6.14, 19.01) <0.001	3.97 (1.86, 8.49) <0.001
Hypertension			
No	73.07 (59.57, 89.62) <0.001	53.26 (42.61, 66.56) <0.001	4.53 (2.99, 6.88) <0.001
Yes	34.53 (18.22, 65.44) <0.001	23.26 (11.94, 45.29) <0.001	4.18 (1.07, 16.32) 0.039
WC (cm)			
Normal	90.58 (71.22, 115.19) <0.001	44.37 (34.14, 57.66) <0.001	4.93 (3.07, 7.89) <0.001
Elevated	21.75 (15.03, 31.47) <0.001	8.61 (5.70, 13.01) <0.001	2.56 (1.19, 5.49) 0.016

Model 1: No covariates were adjusted. Model 2: age and sex were adjusted. Model 3: age, sex, hypertension, smoking status, drinking status, Exercise status, HbA1c, BMI, ALT, AST, GGT, TG, and LDL-C were adjusted. In the subgroup analysis for sex, the model was not adjusted for sex. In the subgroup analysis for age, the model was not adjusted for age. In the subgroup analysis for BMI, the model was not adjusted for BMI. In the subgroup analysis for TG, the model was not adjusted for TG. In the subgroup analysis for hypertension, the model was not adjusted for hypertension. In the subgroup analysis for WC, the model was not adjusted for WC. Elevated WC was defined as ≥90 cm in men and ≥80 cm in women; normal WC was defined as < 90 cm in men and < 80 cm in women.

MASLD, Metabolic dysfunction associated steatotic liver disease; CHG, Cholesterol, high-density lipoprotein, and glucose; BMI, Body mass index; WC, Waist circumference; HbA1c, Glycosylated hemoglobin; ALT, Alanine aminotransferase; AST, Aspartate aminotransferase; GGT, Gamma-glutamyl transpeptidase; TG, Triglycerides; LDL-C, Low-density lipoprotein cholesterol; OR, Odds ratios; CI, Confidence interval.

Sex, age, BMI, TG levels, hypertension status, and WC were further stratified in subgroup analyses. In female participants, CHG was significantly associated with MASLD risk across all three models (Model 1: OR = 39.31, 95% CI: 30.96–49.90; Model 2: OR = 41.79, 95% CI: 32.80–53.26; Model 3: OR = 4.59, 95% CI: 2.89–7.28). A similar pattern of association was observed in male participants (Model 1: OR = 91.27, 95% CI: 61.74–134.91; Model 2: OR = 87.27, 95% CI: 57.85–131.65; Model 3: OR = 3.37, 95% CI: 1.51–7.51). When stratified by age, the positive association between CHG and MASLD risk remained significant in both participants aged <40 years (Model 1: OR = 164.35, 95% CI: 113.55–237.86; Model 2: OR = 109.55, 95% CI: 73.67–162.90; Model 3: OR = 3.95, 95% CI: 1.80–8.64) and those aged ≥40 years (Model 1: OR = 53.61, 95% CI: 42.63–67.42; Model 2: OR = 35.27, 95% CI: 27.63–45.02; Model 3: OR = 4.82, 95% CI: 3.02–7.69).

For BMI stratification, significant positive associations were identified in both participants with BMI < 25 kg/m^2^ (Model 3: OR = 5.83, 95% CI: 3.52–9.65) and those with BMI ≥ 25 kg/m^2^ (Model 3: OR = 2.49, 95% CI: 1.31–4.71). Similarly, TG stratification revealed consistent positive links between CHG and MASLD risk in individuals with TG < 1.7 mmol/L (Model 3: OR = 8.19, 95% CI: 5.48–12.25) and those with TG ≥ 1.7 mmol/L (Model 3: OR = 3.97, 95% CI: 1.86–8.49).

Regardless of hypertension status, CHG was positively associated with MASLD risk in both normotensive (Model 3: OR = 4.53, 95% CI: 2.99–6.88) and hypertensive participants (Model 3: OR = 4.18, 95% CI: 1.07–16.32). Stratification by WC also yielded consistent results, with significant positive associations observed in participants with normal WC (Model 3: OR = 4.93, 95% CI: 3.07–7.89) and elevated WC (Model 3: OR = 2.56, 95% CI: 1.19–5.49). Collectively, these subgroup analyses demonstrate that the positive association between CHG and MASLD risk is robust and consistent across key demographic and clinical subgroups.

### 3.3 Association between CHG and BRI

As presented in Table 3, linear regression analyses revealed a strong positive association between CHG and BRI across all three models (all *p* < 0.001). In the unadjusted Model 1, each unit increase in CHG was associated with a *β* value of 1.28 (95% CI: 1.24–1.32). After adjustment for age and sex (Model 2), the *β* value was 1.32 (95% CI: 1.27–1.36). In the fully adjusted Model 3 (adjusted for age, sex, hypertension, smoking, drinking, Exercise status, HbA1c, ALT, AST, GGT, TG, and LDL-C), the *β* value remained statistically significant at 1.14 (95% CI: 1.06–1.21).

**Table 3:** Association between CHG and BRI.

Variable	Model 1 β (95% CI), *p*-value	Model 2 *β* (95% CI), *p*-value	Model 3 *β* (95% CI), *p*-value
CHG	1.28 (1.24, 1.32) < 0.001	1.32 (1.27, 1.36) < 0.001	1.14 (1.06, 1.21) < 0.001
CHG quartile			
Q1 (4.03–4.85)	Reference	Reference	Reference
Q2 (4.85–5.08)	0.30 (0.26, 0.33) < 0.001	0.29 (0.25, 0.33) < 0.001	0.26 (0.23, 0.30) < 0.001
Q3 (5.08–5.34)	0.69 (0.65, 0.72) < 0.001	0.69 (0.65, 0.73) < 0.001	0.57 (0.53, 0.62) < 0.001
Q4 (5.34–6.40)	1.12 (1.08, 1.16) < 0.001	1.14 (1.09, 1.18) < 0.001	0.83 (0.77, 0.88) < 0.001
*p* for trend	<0.001	<0.001	<0.001

Model 1: No covariates were adjusted. Model 2: age and sex were adjusted. Model 3: age, sex, hypertension, smoking status, drinking status, Exercise status, HbA1c, ALT, AST, GGT, TG, and LDL-C were adjusted.

CHG, Cholesterol, high-density lipoprotein, and glucose; BRI, Body roundness index; HbA1c, Glycosylated hemoglobin; ALT, Alanine aminotransferase; AST, Aspartate aminotransferase; GGT, Gamma-glutamyl transpeptidase; TG, Triglycerides; LDL-C, Low-density lipoprotein cholesterol; β, Standardised regression coefficient; CI, Confidence interval.

When CHG was analysed as a categorical variable by quartiles (Q1 as reference), a significant dose–response relationship was observed between CHG quartiles and BRI in all models (all *p* for trend < 0.001). In Model 3, the *β* values were 0.26 (95% CI: 0.23–0.30) for Q2, 0.57 (95% CI: 0.53–0.62) for Q3, and 0.83 (95% CI: 0.77–0.88) for Q4.

### 3.4 Relationship between BRI and MASLD

Multivariate logistic regression was performed to assess the association between BRI and MASLD risk, with results shown in Table 4. BRI was positively associated with MASLD risk in all three models. In unadjusted Model 1, a per-unit increase in BRI was associated with an OR of 1.66 (95% CI: 1.59–1.73). After adjustment for age and sex in Model 2, the OR was 1.85 (95% CI: 1.76–1.93). In the fully adjusted Model 3, the OR was 1.46 (95% CI: 1.37–1.55).

**Table 4:** Association between BRI and the MASLD risk.

	Model 1 OR (95% CI), *p*-value	Model 2 OR (95% CI), *p*-value	Model 3 OR (95% CI), *p*-value
BRI	1.66 (1.59, 1.73) < 0.001	1.85 (1.76, 1.93) < 0.001	1.46 (1.37, 1.55) < 0.001
BRI quartile			
Q1 (0.63–2.06)	Reference	Reference	Reference
Q2 (2.06–2.60)	2.99 (2.09, 3.89) < 0.001	2.81 (1.91, 3.71) < 0.001	2.48 (1.58, 3.39) < 0.001
Q3 (2.60–3.22)	4.80 (3.92, 5.69) < 0.001	4.58 (3.69, 5.46) < 0.001	3.89 (3.00, 4.78) < 0.001
Q4 (3.22–10.39)	6.24 (5.36, 7.12) < 0.001	6.20 (5.31, 7.08) < 0.001	5.16 (4.27, 6.05) < 0.001
*p* for trend	<0.001	<0.001	<0.001

Model 1: No covariates were adjusted. Model 2: age and sex were adjusted. Model 3: age, sex, hypertension, smoking status, drinking status, Exercise status, HbA1c, ALT, AST, GGT, TG, and LDL-C were adjusted.

MASLD, Metabolic dysfunction associated steatotic liver disease; BRI, Body roundness index; HbA1c, Glycosylated hemoglobin; ALT, Alanine aminotransferase; AST, Aspartate aminotransferase; GGT, Gamma-glutamyl transpeptidase; TG, Triglycerides; LDL-C, Low-density lipoprotein cholesterol; OR, Odds ratios; CI, Confidence interval.

When stratified by BRI quartiles (Q1: 0.63–2.06, Q2: 2.06–2.60, Q3: 2.60–3.22, Q4: 3.22–10.39) with Q1 as reference, MASLD risk increased in a significant dose–response manner with ascending BRI quartiles (all *p* for trend <0.001). In Model 3, compared with Q1, the ORs were 2.48 (95% CI: 1.58–3.39) for Q2, 3.89 (95% CI: 3.00–4.78) for Q3, and 5.16 (95% CI: 4.27–6.05) for Q4.

### 3.5 Nonlinear relationship analysis

**Figure 2:**
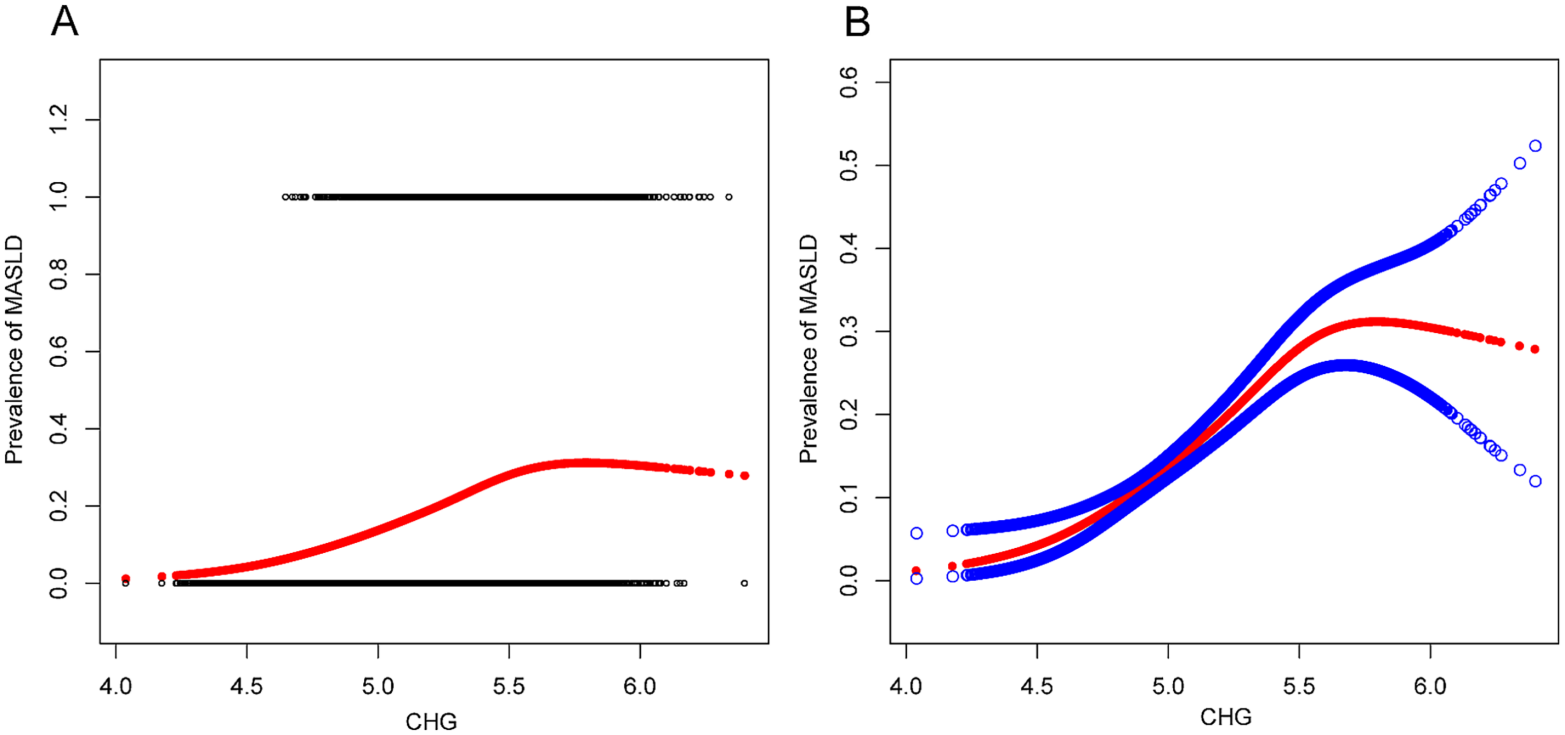
Associations between CHG and prevalence of MASLD. **(A)** and **(B)**: Associations between CHG and prevalence of MASLD. The solid red line represents the smooth curve fit between variables. Blue bands represent the 95% confidence interval from the fit. They were adjusted for age, sex, hypertension, smoking status, drinking status, Exercise status, HbA1c, BMI, ALT, AST, GGT, TG, and LDL-C. MASLD, Metabolic dysfunction-associated steatotic liver disease; CHG, Cholesterol, high-density lipoprotein, and glucose; BMI, Body mass index; HbA1c, Glycosylated hemoglobin; ALT, Alanine aminotransferase; AST, Aspartate aminotransferase; GGT, Gamma-glutamyl transpeptidase; TG, Triglycerides; LDL-C, Low-density lipoprotein cholesterol.

**Figure 3:**
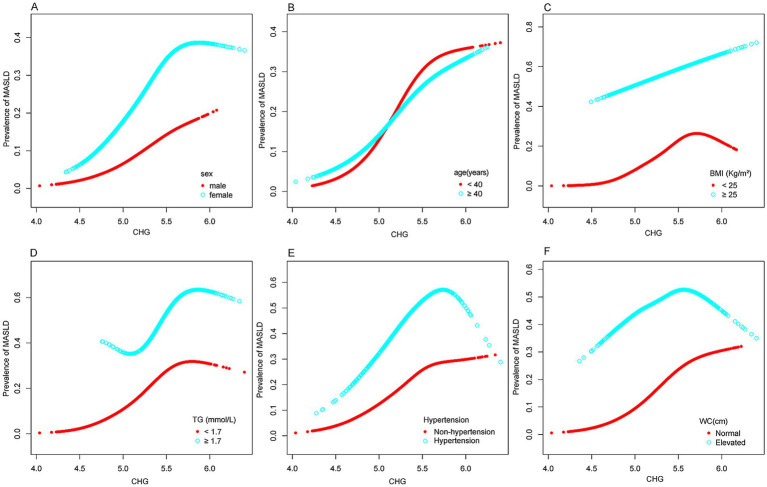
Associations between CHG and the prevalence of MASLD by sex **(A)**, age **(B)**, BMI **(C)**, TG **(D)**, hypertension **(E)**, and WC **(F)**. They were adjusted for age, sex, hypertension, smoking status, drinking status, Exercise status, HbA1c, BMI, ALT, AST, GGT, TG, and LDL-C. In subgroup analyses, the model was not adjusted for the classified variables. MASLD, Metabolic dysfunction-associated steatotic liver disease; CHG, Cholesterol, high-density lipoprotein, and glucose; BMI, Body mass index; WC, Waist circumference; HbA1c, Glycosylated hemoglobin; ALT, Alanine aminotransferase; AST, Aspartate aminotransferase; GGT, Gamma-glutamyl transpeptidase; TG, Triglycerides; LDL-C, Low-density lipoprotein cholesterol.

Smooth curve fitting was performed to examine the nonlinear relationship between CHG and MASLD. A nonlinear positive association between CHG and MASLD risk was observed in the total study population (Figure 2). Subgroup analyses stratified by age, sex, BMI, TG, hypertension, and WC further confirmed consistent nonlinear patterns across all subgroups (Figure 3).

As shown in Table 5, threshold effect analyses using two-piecewise linear regressions were conducted to determine inflexion points. The inflection point was 5.52 in all participants, 5.57 in women, 5.27 in participants aged <40 years, 5.50 in those with BMI < 25 kg/m^2^, 5.49 in subjects with TG < 1.7 mmol/L, 5.51 in non-hypertensive individuals, and 5.46 in participants with normal WC. When CHG levels were below the inflection point, each unit increase in CHG was significantly associated with an increased prevalence of MASLD (all *p* < 0.01). When CHG levels exceeded the inflection point, no significant dose–response association was observed, suggesting a saturation effect. Log-likelihood ratio tests further indicated that the two-piecewise linear model provided a significantly better fit than the standard linear model (all *p* < 0.01).

**Table 5:** Threshold effect analysis of CHG on the prevalence of MASLD across sex, age, BMI, TG, hypertension, and WC using a two-piecewise linear regression model.

Prevalence of MASLD	Adjusted OR (95% CI), *p*-value
All participants	
Fitting by the standard linear model	4.46 (3.00, 6.64), <0.001
Fitting by the two-piecewise linear model	
Inflection point	5.52
CHG < 5.52	7.52 (4.74, 11.92), <0.001
CHG > 5.52	0.86 (0.39, 1.93), 0.718
Log likelihood ratio	<0.001
Women	
Fitting by the standard linear model	4.59 (2.89, 7.28), <0.001
Fitting by the two-piecewise linear model	
Inflection point	5.57
CHG < 5.57	7.46 (4.36, 12.76), <0.001
CHG > 5.57	0.88 (0.32, 2.37), 0.794
Log likelihood ratio	<0.001
Age <40 (years)	
Fitting by the standard linear model	3.95 (1.80, 8.64), 0.006
Fitting by the two-piecewise linear model	
Inflection point	5.27
CHG < 5.27	17.37 (4.96, 60.85), <0.001
CHG > 5.27	1.22 (0.42, 3.53), 0.707
Log likelihood ratio	<0.001
BMI < 25 (kg/m^2^)	
Fitting by the standard linear model	5.83 (3.52, 9.65), <0.001
Fitting by the two-piecewise linear model	
Inflection point	5.50
CHG < 5.50	13.14 (7.15, 24.13), <0.001
CHG > 5.50	0.66 (0.24, 1.77), 0.406
Log likelihood ratio	<0.001
TG < 1.7 (mmol/L)	
Fitting by the standard linear model	8.19 (5.48, 12.25), <0.001
Fitting by the two-piecewise linear model	
Inflection point	5.49
CHG < 5.49	13.89 (8.58, 22.46), <0.001
CHG > 5.49	1.18 (0.43, 3.22), 0.744
Log likelihood ratio	< 0.001
Non-hypertension	
Fitting by the standard linear model	4.53 (2.99, 6.88), <0.0001
Fitting by the two-piecewise linear model	
Inflection point	5.51
CHG < 5.51	7.73 (4.74, 12.60), <0.001
CHG > 5.51	0.93 (0.41, 2.15), 0.871
Log likelihood ratio	<0.001
Normal WC	
Fitting by the standard linear model	4.93 (3.07, 7.89), <0.001
Fitting by the two-piecewise linear model	
Inflection point	5.46
CHG < 5.46	9.36 (5.15, 17.03), <0.001
CHG > 5.46	1.46 (0.64, 3.29), 0.367
Log likelihood ratio	<0.001

Age, sex, hypertension, smoking status, drinking status, Exercise status, HbA1c, BMI, ALT, AST, GGT, TG, and LDL-C were adjusted. In the subgroup analysis for sex, the model was not adjusted for sex. In the subgroup analysis for age, the model was not adjusted for age. In the subgroup analysis for BMI, the model was not adjusted for BMI. In the subgroup analysis for TG, the model was not adjusted for TG. In the subgroup analysis for hypertension, the model was not adjusted for hypertension. In the subgroup analysis for WC, the model was not adjusted for WC. Elevated WC was defined as ≥90 cm in men and ≥80 cm in women; normal WC was defined as <90 cm in men and <80 cm in women.

MASLD, Metabolic dysfunction associated steatotic liver disease; CHG, Cholesterol, high-density lipoprotein, and glucose; BMI, Body mass index; WC, Waist circumference; HbA1c, Glycosylated hemoglobin; ALT, Alanine aminotransferase; AST, Aspartate aminotransferase; GGT, Gamma-glutamyl transpeptidase; TG, Triglycerides; LDL-C, Low-density lipoprotein cholesterol; OR, Odds ratios; CI, Confidence interval.

### 3.6 Discriminatory performance of CHG for MASLD

**Figure 4:**
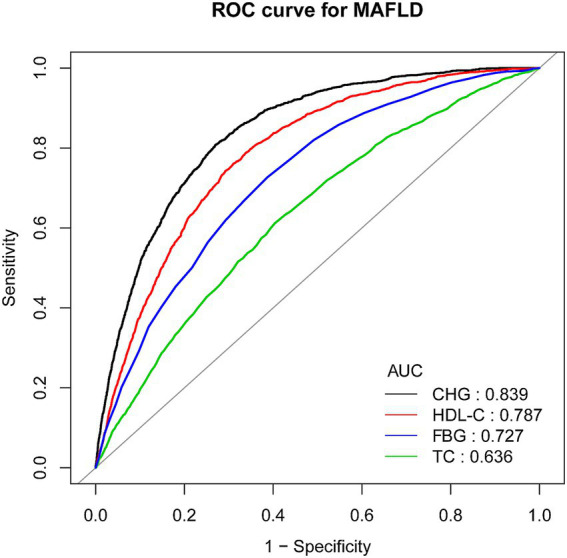
ROC curves comparing the predictive value of CHG with TC, HDL-C, and FBG for MASLD onset, with predictive efficacy assessed by AUC in the general population. MASLD, Metabolic dysfunction-associated steatotic liver disease; CHG, Cholesterol, high-density lipoprotein, and glucose; TC, Total cholesterol; HDL-C, High-density lipoprotein cholesterol; FBG, Fasting blood glucose; ROC, Receiver Operating Characteristic; AUC, Area under the curve.

**Figure 5:**
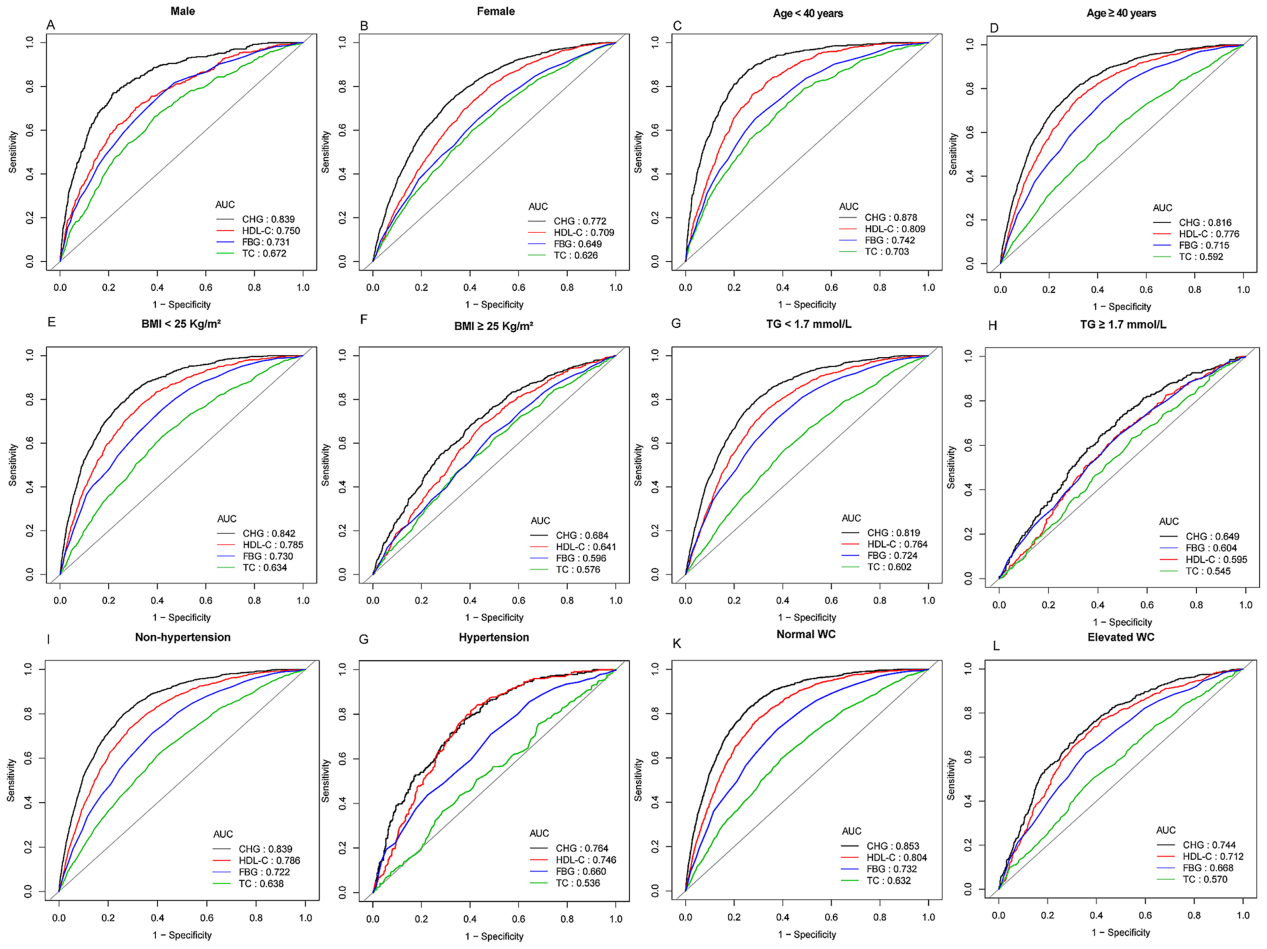
ROC curves comparing the predictive value of CHG with TC, HDL-C, and FBG for MASLD onset, with predictive efficacy assessed by AUC. **(A,B)** Sex stratification: CHG outperformed TC, HDL-C, and FBG in males **(A)** and females **(B)**. **(C,D)** Age stratification: CHG showed better predictive performance in subjects < 40 years **(C)** and ≥ 40 years **(D)**. **(E,F)** BMI stratification: CHG showed higher predictive efficacy in BMI < 25 kg/m^2^ **(E)** and ≥25 kg/m^2^ **(F)**. **(G,H)** TG stratification: CHG outperformed TC, HDL-C, and FBG in TG < 1.7 mmol/L **(G)** and ≥1.7 mmol/L **(H)**. **(I,J)** Hypertension stratification: CHG outperformed TC, HDL-C, and FBG in non-hypertension **(I)** and hypertension **(J)**. **(K,L)** WC stratification: CHG outperformed TC, HDL-C, and FBG in both Normal WC **(K)** and Elevated WC **(L)**. MASLD, Metabolic dysfunction-associated steatotic liver disease; CHG, Cholesterol, high-density lipoprotein, and glucose; BMI, Body mass index; WC, Waist circumference; TG, Triglycerides; TC, Total cholesterol; HDL-C, High-density lipoprotein cholesterol; FBG, Fasting blood glucose; ROC, Receiver operating characteristic; AUC, Area under the curve. Elevated WC was defined as ≥90 cm in men and ≥80 cm in women; normal WC was defined as <90 cm in men and <80 cm in women.

ROC analyses were conducted to evaluate the ability of CHG to identify MASLD in the current study population (Figures 4, 5; Supplementary Table S1). CHG exhibited favorable discriminatory performance in this cohort, with an area under the curve (AUC) of 0.8394 (95% confidence interval [CI]: 0.8310–0.8497), which was significantly higher than the corresponding values for HDL-C (0.7866), FBG (0.7267), and TC (0.6361) (all *p* < 0.01). The optimal cutoff value of CHG for MASLD detection was 5.2298, accompanied by a sensitivity of 0.8074 and a specificity of 0.7305. We further performed stratified analyses to assess the robustness of CHG’s predictive performance across diverse subgroups (Figure 5). In sex-stratified analyses, CHG remained superior to TC, FBG, and HDL-C in both men and women. In male participants, CHG yielded an AUC of 0.8392 (95% CI: 0.8194–0.8590), with an optimal cutoff of 5.1105, sensitivity of 0.7712, and specificity of 0.7806. In female participants, the AUC of CHG was 0.7720 (95% CI: 0.7597–0.7843), with a cutoff of 5.3224, sensitivity of 0.7550, and specificity of 0.6620. By age subgroup, CHG showed higher AUCs of 0.8782 (95% CI: 0.8659–0.8906) in participants aged <40 years and 0.8158 (95% CI: 0.8045–0.8271) in those aged ≥40 years. For BMI subgroups, the AUC values of CHG were 0.8416 (95% CI: 0.8294–0.8537) and 0.6841 (95% CI: 0.6615–0.7068) in participants with BMI < 25 and ≥25 kg/m^2^, respectively. For TG subgroups, CHG achieved an AUC of 0.8188 (95% CI: 0.8084–0.8292) in those with TG < 1.7 mmol/L and 0.6485 (95% CI: 0.6167–0.6803) in those with TG ≥ 1.7 mmol/L. In addition, CHG also presented favourable discriminatory performance in subgroups stratified by hypertension status and WC. In all these subgroups, CHG consistently achieved higher AUC values than TC, FBG, and HDL-C (all *p* < 0.01).

### 3.7 Exploratory mediation analysis of BRI in the CHG–MASLD association

**Figure 6:**
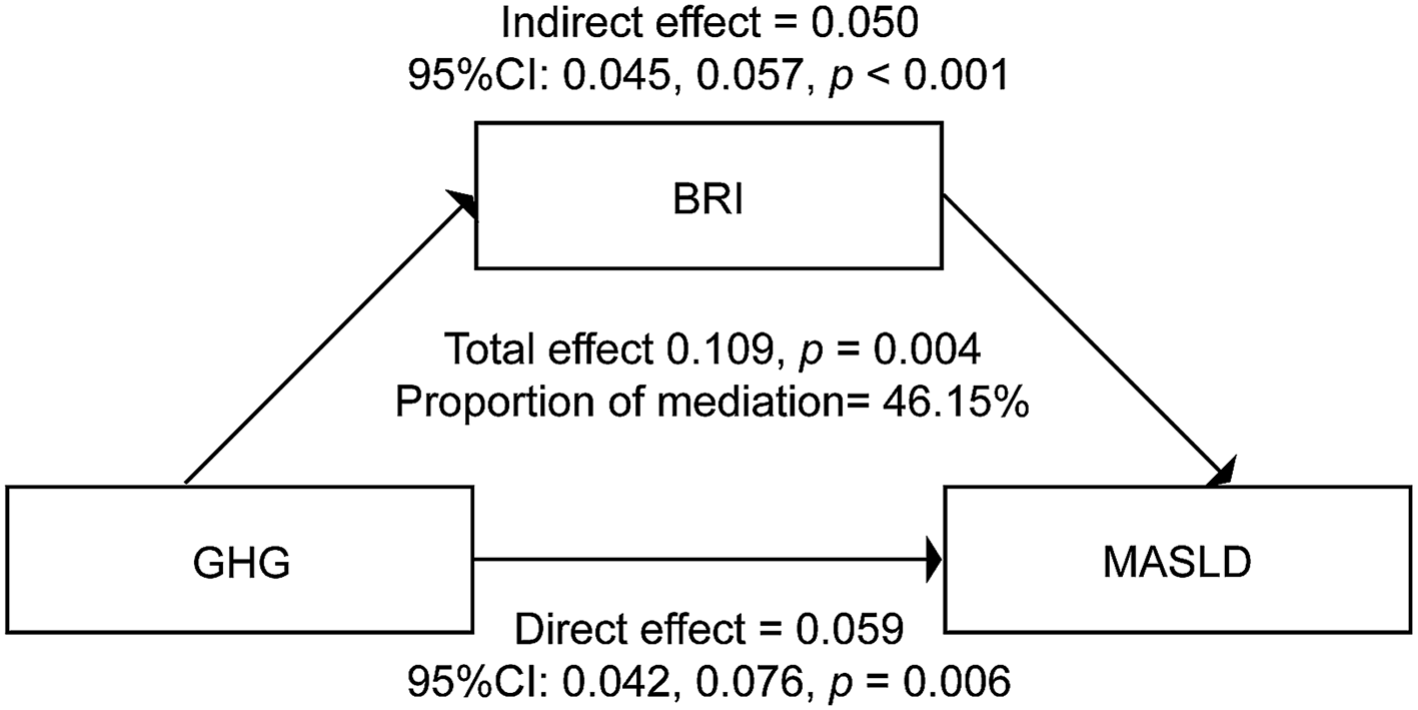
The mediation effect of BRI between CHG and MASLD in the general population. Adjusted for age, sex, hypertension, smoking status, drinking status, Exercise status, HbA1c, ALT, AST, GGT, TG, and LDL-C. MASLD, Metabolic dysfunction-associated steatotic liver disease; CHG, Cholesterol, high-density lipoprotein, and glucose; BRI, Body roundness index; HbA1c, Glycosylated hemoglobin; ALT, Alanine aminotransferase; AST, Aspartate aminotransferase; GGT, Gamma-glutamyl transpeptidase; TG, Triglycerides; LDL-C, Low-density lipoprotein cholesterol.

**Figure 7:**
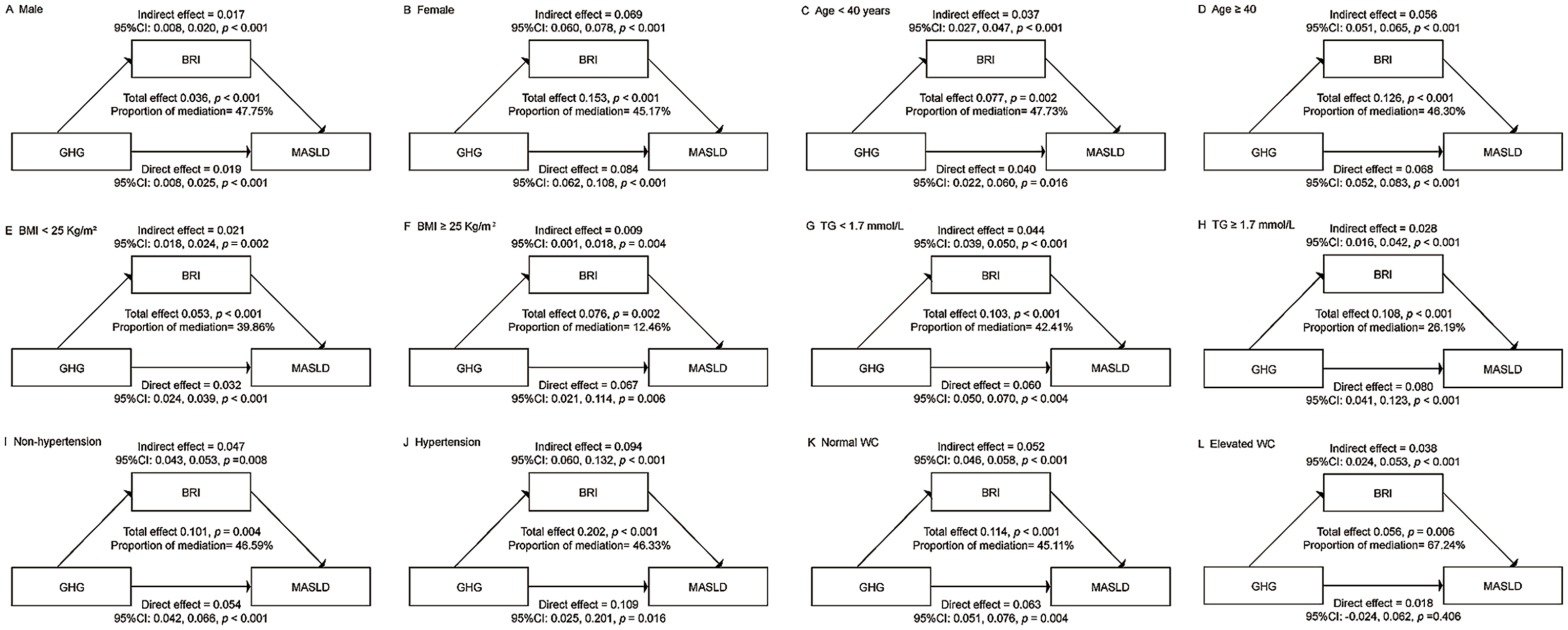
The mediation effect of BRI between CHG and MASLD by sex **(A,B)**, age **(C,D)**, BMI **(E,F)**, TG **(G,H)**, hypertension **(I,J)**, and WC **(K,L)**. Adjusted for age, sex, hypertension, smoking status, drinking status, Exercise status, HbA1c, ALT, AST, GGT, TG, and LDL-C. In subgroup analyses, the model was not adjusted for the classified variables. MASLD, Metabolic dysfunction-associated steatotic liver disease; CHG, Cholesterol, high-density lipoprotein, and glucose; BRI, Body roundness index; BMI, Body mass index; WC, Waist circumference; HbA1c, Glycosylated hemoglobin; ALT, Alanine aminotransferase; AST, Aspartate aminotransferase; GGT, Gamma-glutamyl transpeptidase; TG, Triglycerides; LDL-C, Low-density lipoprotein cholesterol. Elevated WC was defined as ≥90 cm in men and ≥80 cm in women; normal WC was defined as <90 cm in men and <80 cm in women.

Given the observed associations among CHG, BRI, and MASLD, we performed exploratory mediation analysis to decompose the total association into direct and indirect statistical components. The conceptual model and path coefficients are illustrated in Figures 6, 7, with detailed results fully reported in Supplementary Table S2. All analyses were adjusted for age, sex, hypertension, smoking status, drinking status, exercise status, HbA1c, ALT, AST, GGT, TG, and LDL-C, except in the respective subgroup analyses, where the stratification variable was not adjusted for.

In the total study population, BRI potentially and significantly mediated the association between CHG and MASLD. The total effect of CHG on MASLD was 0.109 (95% CI: 0.096–0.123, *p* = 0.004). This effect was decomposed into a significant indirect effect through BRI of 0.050 (95% CI: 0.045–0.057, *p* < 0.001) and a significant direct effect of 0.059 (95% CI: 0.042–0.076, *p* = 0.006). The mediation proportion attributable to BRI was 46.15% (*p* = 0.004).

Subgroup analyses further elucidated this potential mediating pathway across diverse populations. A significant mediation effect of BRI was consistently observed across all subgroups (all *p* for mediation <0.01), though the proportion mediated varied. The mediation proportion was similar between men (47.75%) and women (45.17%), and between participants aged <40 years (47.73%) and those aged ≥40 years (46.30%). Notably, the proportion mediated was lower in participants with BMI ≥ 25 kg/m^2^ (12.46%) and TG ≥ 1.7 mmol/L (26.19%) compared to their counterparts with BMI < 25 kg/m^2^ (39.86%) and TG < 1.7 mmol/L (42.41%). In subgroups stratified by hypertension status and WC, the mediation proportions were 46.59% (non-hypertensive) vs. 46.33% (hypertensive), and 45.11% (normal WC) vs. 67.24% (elevated WC), respectively.

### 3.8 Sensitivity analysis for incorporation bias

When ultrasound-diagnosed isolated hepatic steatosis was used as the outcome, the independent positive association between CHG and hepatic steatosis remained statistically significant after full adjustment (OR = 2.94, 95% CI: 2.06–4.20, *p* < 0.001). BRI still exerted a significant partial mediating effect, with a mediation proportion of 58.44%. The direction and statistical significance of all effects were consistent with the primary MASLD outcome analysis. Detailed results are provided in Supplementary Tables S3 and S4.

## 4 Discussion

Using cross-sectional data from the NAGALA cohort of Japanese adults without diabetes, we demonstrated that elevated CHG was independently and nonlinearly associated with MASLD risk in a dose–response fashion. CHG showed better discriminatory performance for MASLD than conventional single metabolic markers, with robust predictive performance in non-obese and young individuals. Exploratory mediation analysis showed that BRI contributed a statistically significant partial mediating effect (46.15%) to the observed CHG- MASLD association. This finding should be interpreted as a statistical decomposition of associations rather than confirmation of a causal pathway. Notably, the sensitivity analysis using ultrasound-only hepatic steatosis as the outcome replicated both the independent association of CHG and the partial mediating effect of BRI. Although the magnitude of the main association was attenuated as expected after removing overlapping metabolic components, the statistical significance and direction of effects remained unchanged. This indicates that our core findings are not driven by incorporation bias from overlapping metabolic features in the MASLD diagnostic criteria. These findings support the potential utility of CHG as a low-cost indicator for early MASLD risk stratification.

This study rigorously excluded participants with diabetes and impaired glucose tolerance, minimising confounding from hyperglycemia and glucose-lowering medications and yielding a more robust independent association between CHG and MASLD. As a novel composite metabolic marker combining TC, HDL-C, and FBG, CHG captures both impaired reverse cholesterol transport and disrupted glucose homeostasis and is closely linked to IR, T2DM, and cardiovascular disease risk (23, 40, 41). Published evidence indicates that CHG shows a U-shaped nonlinear association with all-cause and cardiovascular mortality in metabolic syndrome, and is also associated with hypertension, stroke, diabetic retinopathy, and other cardiometabolic disorders (40, 42–45). Importantly, a Chinese cohort study by Li et al. (46) confirmed that CHG outperforms conventional metabolic markers in predicting carotid plaque and fatty liver disease.

Recent studies in MASLD have further substantiated the clinical value of CHG. In two prospective cohorts, Zhu et al. (32) reported that higher CHG levels were significantly associated with elevated risks of all-cause and cardiovascular mortality in patients with MASLD, following a typical U-shaped nonlinear pattern. In non-obese participants from China and the Japanese cohort, Chen et al. observed that each 1-unit increase in CHG was associated with a dose-dependent rise in MASLD risk. CHG yielded an AUC exceeding 0.80 for MASLD identification, qualifying it as an independent predictor of MASLD incidence and a poor prognostic factor (47). Overall, our results align with existing literature, reinforcing the strong link between elevated CHG and increased MASLD risk, as well as the favourable discriminatory power of CHG. Our study advances prior work in three key ways: (1) we included only non-diabetic individuals to avoid diabetic confounding; (2) we first demonstrate BRI mediates the CHG-MASLD association; (3) we confirm CHG performs well in non-obese and young individuals.

We also identified a positive nonlinear association between CHG and MASLD risk, characterised by a clear threshold effect. Smooth curve fitting and piecewise regression revealed that MASLD prevalence rose sharply with increasing CHG below the inflection point. In contrast, no significant dose–response association was detected beyond this threshold, indicating a saturation effect. Pathophysiological mechanisms, detection limitations, and population characteristics can explain this pattern. The strong discriminatory performance of CHG for MASLD stems from its integration of multiple pathological pathways related to IR, chronic inflammation, and oxidative stress (23, 40, 42). At high CHG levels, severe glycolipid metabolic disturbance, combined with insulin resistance, disordered cholesterol metabolism, and impaired glucose homeostasis, may saturate hepatic steatosis, such that further elevations in CHG no longer augment disease risk. Of note, individuals with extremely high CHG often present with complex metabolic comorbidities, which may introduce residual confounding. Clinically, these findings suggest that early metabolic intervention in individuals with CHG levels below the inflection point may enable more precise prevention and management of MASLD.

Findings from ROC analysis indicate promising discriminatory capacity of CHG for MASLD in this Japanese non-diabetic population. CHG showed significantly better discriminatory ability for MASLD than TC, FBG, or HDL-C alone, and remained stable across all subgroups. Notably, CHG maintained a high AUC in non-obese and young individuals, suggesting its potential to complement conventional obesity indices and single metabolic markers in populations easily missed by routine screening. Conventional BMI and WC are prone to miss MASLD cases in non-obese individuals; CHG retains high discriminative efficacy among participants with BMI < 25 kg/m^2^, indicating potential value in filling this screening gap.

Since the current analysis is based on a single cross-sectional cohort and lacks prospective longitudinal validation and external verification in independent populations, these results should be interpreted as preliminary evidence. Larger prospective multi-center studies are warranted to validate its performance before CHG can be recommended as a routine screening tool in primary care and community settings.

To further delineate the pathophysiological mechanisms underpinning the association between CHG and MASLD, we conducted mediation analyses with the BRI as the mediating variable. In the overall study cohort, BRI accounted for 46.15% of the total effect of CHG on MASLD risk, indicating that visceral adiposity, as quantified by BRI, functions as a pivotal intermediate pathway linking systemic glucolipid metabolic dysregulation to hepatic steatosis. Metabolic disturbances caused by high CHG impair insulin signalling and adipose lipid homeostasis, promoting systemic insulin resistance and visceral fat accumulation (32, 48, 49). Increased free fatty acid (FFA) flux into the liver via the portal circulation stimulates *de novo* hepatic lipogenesis while suppressing fatty acid oxidation, ultimately leading to aberrant triglyceride accumulation in hepatocytes (50–53). Concomitantly, dysfunctional visceral adipose tissue hypersecretes pro-inflammatory cytokines such as tumor necrosis factor-*α* (TNF-α) and interleukin-6 (IL-6), while diminishing the synthesis and release of anti-inflammatory adipokines (e.g., adiponectin). This imbalance amplifies hepatic inflammation and oxidative stress, synergistically driving the initiation and progression of MASLD (54–56).

CHG integrates two core metabolic axes implicated in MASLD pathogenesis: glucotoxicity and lipotoxicity. Elevated TC and reduced HDL-C not only reflect atherosclerotic burden but also correlate with systemic oxidative stress, immune dysregulation, and impaired hepatic lipid secretion—all of which exacerbate IR through multiple interconnected biological pathways (57). Furthermore, cholesterol metabolism and hepatic IR exhibit a close bidirectional regulatory crosstalk (58). Enhanced hepatic lipotoxicity, intracellular cholesterol accumulation, and defective lipoprotein processing collectively impair the IRS-1/PI3K/Akt signalling cascade, thereby accelerating the progression of glucose metabolic disorders (59).

The heterogeneity in the mediating effect of BRI observed in this study is highly consistent with the multiple-hit hypothesis of MASLD pathogenesis. The mediating proportion of BRI was significantly reduced to 12.46 and 26.19% in overweight/obese (BMI ≥ 25 kg/m^2^) and hypertriglyceridemic (TG ≥ 1.7 mmol/L) individuals, respectively. In subjects with severe pre-existing metabolic disorders, the liver undergoes simultaneous and intensive multiple hits, and CHG contributes to MASLD mainly through direct hepatocellular injury pathways independent of BRI. Severe hyperglycemia and atherogenic dyslipidemia, when associated with elevated CHG, directly induce hepatocellular lipotoxicity, cholesterol overload, endoplasmic reticulum stress, mitochondrial dysfunction, and impaired insulin signalling, thereby promoting intrahepatic lipid deposition (60–62). In this context, direct hepatic injury becomes the dominant driver, weakening the mediating role of visceral adiposity, as represented by BRI. In contrast, the mediating proportion of BRI was significantly higher (39.86 and 42.41%, respectively) in non-overweight/non-obese (BMI < 25 kg/m^2^) and normotriglyceridemic (TG < 1.7 mmol/L) individuals. This indicates that, in individuals with early or mild metabolic abnormalities, elevated CHG mainly promotes visceral adiposity accumulation through metabolic disturbances, which, in turn, leads to hepatic steatosis. Importantly, the mediating proportion of BRI reached 67.24% in participants with abdominal obesity (elevated WC), further confirming that visceral adiposity is the key mediator underlying the increased risk of MASLD associated with high CHG levels in individuals with abdominal obesity. This finding significantly enhances the mechanistic insights of the present study and holds important clinical implications. For non-obese high-risk individuals with mild metabolic abnormalities, early interventions targeting visceral fat reduction may be more efficacious. Conversely, for those with severe metabolic disorders (e.g., obesity, hypertriglyceridemia), greater emphasis should be placed on direct metabolic modulation to block the direct lipotoxic and glucotoxic effects of CHG on hepatocytes, thereby enabling more precise prevention of MASLD onset and progression.

### 4.1 Study strengths and limitations

Our study has three major strengths. First, the study design is rigorous: we enrolled a community-based non-diabetic population, avoiding confounding from glucose metabolic disorders and glucose-lowering medications, thereby allowing a more accurate assessment of the independent association between CHG and MASLD. Second, the mechanistic insights are novel: by using BRI as a potential mediator, we demonstrate, for the first time, the key role of visceral fat accumulation in CHG-related MASLD, refining the pathophysiological cascade by which glycolipid metabolic disorders induce hepatic steatosis. Third, the translational potential is clear: we defined a threshold effect for CHG, providing a quantitative tool for early MASLD risk stratification. Since CHG is derived from routine laboratory tests, it is simple, non-invasive, and easy to implement, with high potential for clinical application and public health translation.

Several limitations warrant mention. First, owing to the cross-sectional design, all variables were measured at a single time point, which precludes confirmation of the temporal sequence (CHG → BRI → MASLD) required for causal inference. The mediation analysis performed in this study is exploratory in nature and reflects only a statistical decomposition of observed associations; it cannot confirm that BRI acts as a causal mediator, nor can it rule out potential confounding or reverse causality. Prospective cohort studies are needed to validate the causal direction of the pathway. Second, MASLD was diagnosed by abdominal ultrasound, which cannot distinguish simple steatosis from MASH or assess hepatic fibrosis stage, thereby introducing potential misclassification bias. Third, we did not adjust for BMI in the core mediation model to avoid collinearity with BRI and over-adjustment bias, which represents a methodological trade-off to accurately estimate the mediating role of visceral adiposity. Although this approach may theoretically leave residual confounding from overall adiposity, it aligns with our primary research question and represents standard practice in the field. Fourth, although we adjusted for major established confounders, residual confounding cannot be fully excluded due to unmeasured or insufficiently characterized factors, including dietary intake patterns, comprehensive medication history, genetic susceptibility (e.g., PNPLA3 polymorphisms), socioeconomic status, detailed intensity of physical activity, and longitudinal trajectories of body weight and metabolic state. These factors may modulate both circulating metabolic profiles and MASLD risk, and their unavailability could have subtly influenced the magnitude of the observed associations and mediation estimates. Fifth, our study population comprised Japanese non-diabetic adults, and generalizability to other ethnicities and individuals with diabetes requires further validation. Sixth, the discriminatory performance and optimal cutoff value of CHG were derived from a single Japanese cross-sectional cohort and only internally validated via 1,000 bootstrap resampling. Lack of prospective longitudinal validation and external validation in independent multi-ethnic populations means it is premature to regard CHG as an established screening biomarker for MASLD. Further large-scale prospective studies are needed to verify its generalizability and clinical utility before widespread translation. Despite these limitations, the large sample, rigorous statistics, and comprehensive subgroup analyses support the reliability of our findings.

## 5 Conclusion

In this large cross-sectional study of non-diabetic Japanese adults, the CHG showed an independent, nonlinear, and positive association with MASLD risk, partially mediated by the BRI. CHG demonstrated good discriminatory performance for MASLD, particularly in non-obese and younger individuals. As an easy-to-obtain, non-invasive, and low-cost biomarker derived from routine biochemistry, CHG holds promise for early MASLD risk stratification and screening in community and primary care settings.

## Data Availability

Publicly available datasets were analyzed in this study. This data can be found at: the data that support the findings of this study are available from Dryad Digital Repository at https://datadryad.org/stash/dataset/doi:10.5061%2Fdryad.8q0p192.

## Footnotes

1
https://doi.org/10.5061/dryad.8q0p192

## Glossary

MASLD: metabolic dysfunction-associated steatotic liver disease
BMI: body mass index
WC: waist circumference
SBP: systolic blood pressure
DBP: diastolic blood pressure
ALT: alanine aminotransferase
AST: aspartate aminotransferase
GGT: gamma-glutamyl transpeptidase
HbA1c: glycosylated hemoglobin
FBG: fasting blood glucose
TG: triglycerides
TC: total cholesterol
HDL-C: high-density lipoprotein cholesterol
LDL-C: low-density lipoprotein cholesterol
CHG: cholesterol, high-density lipoprotein and glucose;
BRI: body roundness index
OR: odds ratios
CI: confidence interval
*β*: standardization regression coefficient
ROC: receiver operating characteristic
AUC: area under curve
