# Supplementary material for: Exploratory mediation analysis of body roundness index in the nonlinear association between CHG index and metabolic dysfunction-associated steatotic liver disease among non-diabetic adults
Source: Front Nutr. 2026 Jul 23;13:1868410. doi: 10.3389/fnut.2026.1868410 (PMC13446269; doi:10.3389/fnut.2026.1868410)
Supplement: Supplementary file 1 [file Table_1.DOCX]

Supplementary Material

**Supplementary Tables**

**Supplemental Table 1.** The best threshold, sensitivities, specificities, and AUC of each parameter for screening MASLD in the general population and subgroup analysis for sex, age, BMI, TG, Hypertension, and WC.

|  | **AUC** | **95% CI** | **Best threshold** | **Specificity** | **Sensitivity** |
| --- | --- | --- | --- | --- | --- |
| **General population** | | | | | |
| CHG | 0.8394 | 0.8310-0.8497 | 5.2298 | 0.7305 | 0.8074 |
| HDL-C | 0.7866* | 0.7768-0.7964 | 50.1500 | 0.6895 | 0.7609 |
| TC | 0.6361* | 0.6234-0.6488 | 201.5000 | 0.5961 | 0.6119 |
| FBG | 0.7267* | 0.7155-0.7379 | 93.5000 | 0.6140 | 0.7259 |
| **Subgroup analysis stratified by sex** | | | | | |
| **Men** | | | | | |
| CHG | 0.8392 | 0.8194-0.8590 | 5.1105 | 0.7806 | 0.7712 |
| HDL-C | 0.7503* | 0.7255-0.7750 | 56.3500 | 0.6892 | 0.7018 |
| TC | 0.6716* | 0.6441-0.6990 | 202.5000 | 0.6100 | 0.6632 |
| FBG | 0.7311* | 0.7061-0.7561 | 90.5000 | 0.5870 | 0.7609 |
| **Women** | | | | | |
| CHG | 0.7720 | 0.7597-0.7843 | 5.3224 | 0.6620 | 0.7550 |
| HDL-C | 0.7094* | 0.6960-0.7227 | 47.2500 | 0.5857 | 0.7362 |
| TC | 0.6265* | 0.6113-0.6416 | 201.5000 | 0.5909 | 0.5995 |
| FBG | 0.6489* | 0.6340-0.6638 | 95.5000 | 0.5680 | 0.6496 |
| **Subgroup analysis stratified by age (years)** | | | | | |
| **< 40** | | | | | |
| CHG | 0.8782 | 0.8659-0.8906 | 5.1580 | 0.7422 | 0.8761 |
| HDL-C | 0.8094* | 0.7939-0.8249 | 49.0500 | 0.7244 | 0.7645 |
| TC | 0.7035* | 0.6825-0.7244 | 187.5000 | 0.5789 | 0.7248 |
| FBG | 0.7417* | 0.7223-0.7611 | 94.5000 | 0.7140 | 0.6514 |
| **≥ 40** | | | | | |
| CHG | 0.8158 | 0.8045-0.8271 | 5.2303 | 0.6847 | 0.8081 |
| HDL-C | 0.7756* | 0.7632-0.7879 | 50.5500 | 0.6781 | 0.7585 |
| TC | 0.5924* | 0.5762-0.6085 | 209.5000 | 0.6010 | 0.5429 |
| FBG | 0.7145* | 0.7007-0.7284 | 93.5000 | 0.5802 | 0.7404 |
| **Subgroup analysis stratified by BMI (kg/m^2^)** | | | | | |
| **< 25** | | | | | |
| CHG | 0.8416 | 0.8294-0.8537 | 5.1631 | 0.6927 | 0.8420 |
| HDL-C | 0.7855* | 0.7709-0.8000 | 51.8500 | 0.6752 | 0.7677 |
| TC | 0.6337* | 0.6148-0.6527 | 199.5000 | 0.5809 | 0.6295 |
| FBG | 0.7302* | 0.7136-0.7468 | 92.5000 | 0.5814 | 0.7526 |
| **≥ 25** | | | | | |
| CHG | 0.6841 | 0.6615-0.7068 | 5.3535 | 0.5937 | 0.6876 |
| HDL-C | 0.6409* | 0.6172-0.6647 | 47.1500 | 0.5450 | 0.6884 |
| TC | 0.5764* | 0.5520-0.6008 | 213.5000 | 0.6511 | 0.4715 |
| FBG | 0.5955* | 0.5713-0.6197 | 95.5000 | 0.5114 | 0.6378 |
| **Subgroup analysis stratified by TG (mmol/L)** | | | | | |
| **< 1.7** | | | | | |
| CHG | 0.8188 | 0.8084-0.8292 | 5.1643 | 0.6932 | 0.8130 |
| HDL-C | 0.7640* | 0.7521-0.7758 | 52.2500 | 0.6604 | 0.7578 |
| TC | 0.6023* | 0.5872-0.6174 | 199.5000 | 0.5860 | 0.5763 |
| FBG | 0.7236* | 0.7104-0.7368 | 93.5000 | 0.6249 | 0.7115 |
| **≥ 1.7** |  |  |  |  |  |
| CHG | 0.6485 | 0.6167-0.6803 | 5.5067 | 0.4981 | 0.7382 |
| HDL-C | 0.5952* | 0.5622-0.6283 | 41.4500 | 0.5077 | 0.6599 |
| TC | 0.5451* | 0.5116-0.5785 | 212.5000 | 0.4617 | 0.6317 |
| FBG | 0.6038* | 0.5713-0.6363 | 95.5000 | 0.5096 | 0.6505 |
| **Subgroup analysis stratified by Hypertension** | | | | | |
| **No** |  |  |  |  |  |
| CHG | 0.8393 | 0.8302-0.8484 | 5.2284 | 0.7368 | 0.7998 |
| HDL-C | 0.7864* | 0.7758-0.7969 | 49.6500 | 0.7101 | 0.7385 |
| TC | 0.6377* | 0.6240-0.6514 | 199.5000 | 0.5796 | 0.6336 |
| FBG | 0.7221* | 0.7100-0.7342 | 93.5000 | 0.6217 | 0.7124 |
| **Yes** |  |  |  |  |  |
| CHG | 0.7640 | 0.7301-0.7979 | 5.2956 | 0.6238 | 0.7747 |
| HDL-C | 0.7461* | 0.7109-0.7813 | 51.2500 | 0.5947 | 0.8179 |
| TC | 0.5362* | 0.4942-0.5781 | 212.5000 | 0.5728 | 0.5062 |
| FBG | 0.6599* | 0.6208-0.6991 | 95.5000 | 0.5146 | 0.7099 |
| **Subgroup analysis stratified by WC** | | | | | |
| **Normal** |  |  |  |  |  |
| CHG | 0.8534 | 0.8435-0.8633 | 5.2284 | 0.7427 | 0.8226 |
| HDL-C | 0.8040* | 0.7925-0.8154 | 50.1500 | 0.6961 | 0.7828 |
| TC | 0.6319* | 0.6157-0.6480 | 201.5000 | 0.6077 | 0.5956 |
| FBG | 0.7324* | 0.7186-0.7463 | 93.5000 | 0.6223 | 0.7290 |
| **Elevated** |  |  |  |  |  |
| CHG | 0.7438 | 0.7212-0.7664 | 5.3008 | 0.6741 | 0.7005 |
| HDL-C | 0.7119* | 0.6881-0.7356 | 51.4500 | 0.5778 | 0.7704 |
| TC | 0.5704* | 0.5440-0.5967 | 212.5000 | 0.6106 | 0.5058 |
| FBG | 0.6680* | 0.6432-0.6927 | 95.5000 | 0.6434 | 0.6189 |

* *P* < 0.01, compared with CHG. **Abbreviations:** MASLD, Metabolic dysfunction associated steatotic liver disease; CHG, Cholesterol, high-density lipoprotein, and glucose; BMI, Body mass index; WC, Waist circumference; FBG, Fasting blood glucose; TC, Total cholesterol; HDL-C, High-density lipoprotein cholesterol; TG, Triglycerides; CI, Confidence interval; ROC, Receiver operating characteristic; AUC, Area under the curve.

**Supplemental Table 2.** Analysis of the mediation by BRI of the associations of CHG with the risk of MASLD

|  | **Mediation effect (95% CI), *P* value** | | |  |
| --- | --- | --- | --- | --- |
|  | Total effect | Indirect effect | Direct effect | Mediation |
| **BRI** | 0.109 (0.096,0.123),  0.004 | 0.050 (0.045,0.057),  < 0.001 | 0.059 (0.042,0.076),  0.006 | 46.15%,  0.004 |
| **Subgroup analysis stratified by sex** | | | | |
| **Men** | 0.036 (0.018,0.042),  < 0.001 | 0.017 (0.008,0.020),  < 0.001 | 0.019 (0.008,0.025),  < 0.001 | 47.75%,  < 0.001 |
| **Women** | 0.153 (0.129,0.178),  < 0.001 | 0.069 (0.060,0.078),  < 0.001 | 0.084 (0.062,0.108),  < 0.001 | 45.17%,  < 0.001 |
| **Subgroup analysis stratified by age (years)** | | | | |
| **< 40** | 0.077 (0.057,0.099),  0.002 | 0.037 (0.027,0.047),  < 0.001 | 0.040 (0.022,0.060),  0.016 | 47.73%,  0.002 |
| **≥ 40** | 0.126 (0.109,0.141),  < 0.001 | 0.056 (0.051,0.065),  < 0.001 | 0.068 (0.052,0.083),  < 0.001 | 46.30%,  < 0.001 |
| **Subgroup analysis stratified by BMI (kg/m^2^)** | | | | |
| **< 25** | 0.053 (0.045,0.060),  < 0.001 | 0.021(0.018,0.024),  0.002 | 0.032 (0.024,0.039),  < 0.001 | 39.86%,  0.002 |
| **≥ 25** | 0.076 (0.030,0.126),  0.002 | 0.009 (0.001,0.018),  0.004 | 0.067 (0.021,0.114),  0.006 | 12.46%,  0.008 |
| **Subgroup analysis stratified by TG (mmol/L)** | | | | |
| **< 1.7** | 0.103 (0.092,0.116),  < 0.001 | 0.044 (0.039,0.050),  < 0.001 | 0.060 (0.050,0.070),  0.004 | 42.41%,  < 0.001 |
| **≥ 1.7** | 0.108 (0.068,0.154),  < 0.001 | 0.028(0.016,0.042),  < 0.001 | 0.080 (0.041,0.123),  < 0.001 | 26.19%,  < 0.001 |
| **Subgroup analysis stratified by Hypertension** | | | | |
| **No** | 0.101 (0.089,0.114),  0.004 | 0.047 (0.043,0.053),  0.008 | 0.054 (0.042,0.066),  < 0.001 | 46.59%,  0.004 |
| **Yes** | 0.202 (0.109,0.297),  < 0.001 | 0.094 (0.060,0.132),  < 0.001 | 0.109 (0.025,0.201),  0.016 | 46.33%,  < 0.001 |
| **Subgroup analysis stratified by WC** | | | | |
| **Normal** | 0.114 (0.103,0.129),  < 0.001 | 0.052 (0.046,0.058),  < 0.001 | 0.063 (0.051,0.076),  0.004 | 45.11%,  < 0.001 |
| **Elevated** | 0.056 (0.014,0.100),  0.006 | 0.038 (0.024,0.053),  < 0.001 | 0.018 (-0.024,0.062),  0.406 | 67.24%,  0.006 |

Age, sex, Hypertension Status, smoking status, drinking status, exercise status, HbA1c, ALT, AST, GGT, TG, and LDL-C were adjusted for. In the subgroup analysis for sex, the model was not adjusted for sex. In the subgroup analysis for age, the model was not adjusted for age. In the subgroup analysis for TG, the model was not adjusted for TG. In the subgroup analysis for Hypertension, the model was not adjusted for Hypertension. In the subgroup analysis for WC, the model was not adjusted for WC. Elevated WC was defined as ≥ 90 cm in men and ≥ 80 cm in women; normal WC was defined as < 90 cm in men and < 80 cm in women.

**Abbreviations:** MASLD, Metabolic dysfunction associated steatotic liver disease; CHG, Cholesterol, high-density lipoprotein, and glucose; BRI, Body roundness index; BMI, Body mass index; WC, Waist circumference; HbA1c, Glycosylated hemoglobin; ALT, Alanine aminotransferase; AST, Aspartate aminotransferase; GGT, Gamma-glutamyl transpeptidase; TG, Triglycerides; LDL-C, Low-density lipoprotein cholesterol; CI, Confidence interval.

**Supplemental Table 3.** Associations between CHG and the risk of hepatic steatosis.

|  | **Model 1 OR (95% CI), *P* value** | **Model 2 OR (95% CI), *P* value** | **Model 3 OR (95% CI), *P* value** |
| --- | --- | --- | --- |
| **CHG** | 51.31 (43.04, 61.16) <0.001 | 34.33 (28.34, 41.59) <0.001 | 2.94 (2.06, 4.20) <0.001 |
| Q1(4.03-4.85) | Reference | Reference | Reference |
| Q2(4.85-5.08) | 3.17 (2.39, 4.21) <0.001 | 2.72 (2.04, 3.61) <0.001 | 1.57 (1.15, 2.14) 0.004 |
| Q3(5.08-5.34) | 11.30 (8.73, 14.64) <0.001 | 8.26 (6.33, 10.77) <0.001 | 2.47 (1.82, 3.36) <0.001 |
| Q4(5.34-6.40) | 39.04 (30.32, 50.27) <0.001 | 25.24 (19.38, 32.86) <0.001 | 3.17 (2.26, 4.46) <0.001 |
| *P* for trend | < 0.001 | < 0.001 | < 0.001 |

Model 1: No covariates were adjusted. Model 2: age and sex were adjusted. Model 3: age, sex, hypertension, smoking status, drinking status, Exercise status, HbA1c, BMI, ALT, AST, GGT, TG, and LDL-C were adjusted.

**Abbreviations:** CHG, Cholesterol, high-density lipoprotein, and glucose; BMI, Body mass index; HbA1c, Glycosylated hemoglobin; ALT, Alanine aminotransferase; AST, Aspartate aminotransferase; GGT, Gamma-glutamyl transpeptidase; TG, Triglycerides; LDL-C, Low-density lipoprotein cholesterol; OR, Odds ratios; CI, Confidence interval.

**Supplemental Table 4.** Mediation analysis of BRI in the association between CHG and ultrasound-diagnosed hepatic steatosis.

|  | **Mediation effect (95% CI), *P* value** | | |  |
| --- | --- | --- | --- | --- |
|  | Total effect | Indirect effect | Direct effect | Mediation |
| **BRI** | 0.106 (0.091,0.123),  0.002 | 0.054 (0.048,0.060),  < 0.001 | 0.052 (0.038,0.069),  0.002 | 58.44%,  0.002 |

Age, sex, Hypertension Status, smoking status, drinking status, exercise status, HbA1c, ALT, AST, GGT, TG, and LDL-C were adjusted for.

**Abbreviations:**CHG, Cholesterol, high-density lipoprotein, and glucose; BRI, Body roundness index; HbA1c, Glycosylated hemoglobin; ALT, Alanine aminotransferase; AST, Aspartate aminotransferase; GGT, Gamma-glutamyl transpeptidase; TG, Triglycerides; LDL-C, Low-density lipoprotein cholesterol; CI, Confidence interval.
